# Differences in HIV risk and healthcare engagement factors in Filipinx transgender women and cisgender men who have sex with men who reported being HIV negative, HIV positive or HIV unknown

**DOI:** 10.1002/jia2.25582

**Published:** 2020-08-26

**Authors:** Arjee J Restar, Harry Jin, Adedotun Ogunbajo, Alexander Adia, Anthony Surace, Laufred Hernandez, Susan Cu‐Uvin, Don Operario

**Affiliations:** ^1^ Department of Behavioral and Social Sciences Brown University School of Public Health Providence RI USA; ^2^ The Philippine Health Initiative for Research, Service, and Training Brown University Global Health Initiative Providence RI USA; ^3^ amfAR Foundation of AIDS Research Washington DC USA; ^4^ Department of Epidemiology Brown University School of Public Health Providence RI USA; ^5^ Department of Behavioral Sciences University of Philippines Manila Manila Philippines; ^6^ Providence‐Boston Center for AIDS Research Providence RI USA; ^7^ Department of Medicine Miriam Hospital Providence RI USA

**Keywords:** transgender people, men who have sex with men, HIV epidemiology, Asia, key and vulnerable populations, testing, HIV status, Philippines

## Abstract

**Introduction:**

Understanding HIV risk and healthcare engagement of at‐risk individuals by HIV status is vital to informing HIV programmes in settings where the HIV epidemic is rapidly expanding like the Philippines. This study examined differences in HIV risk and healthcare engagement factors among Filipinx transgender women and cisgender men who have sex with men (trans‐WSM and cis‐MSM respectively) who self‐reported being HIV negative, HIV positive or HIV unknown.

**Methods:**

Between 2018 and 2019, we conducted Project #ParaSaAtin, an online cross‐sectional survey that examined the structural, social and behavioural factors impacting HIV services among Filipinx trans‐WSM and cis‐MSM (n = 318). We performed multinomial regression procedures to determine factors associated with HIV status (with HIV‐negative referent). Co‐variates included participant demographics, experiences of social marginalization, HIV risk, healthcare engagement and alcohol and substance problems.

**Results:**

Self‐reported HIV status of the sample was as follows: 38% HIV negative, 34% HIV positive and 28% HIV unknown. Relative to HIV‐negative respondents, HIV‐positive respondents were more likely to be older (25‐ to 29‐year‐old adjusted risk ratio [aRRR]=5.08, 95% Confidence Interval [95% CI] = 1.88 to 13.72; 30‐ to 34‐year‐old aRRR = 4.11, 95% CI = 1.34 to 12.58; and 35 + years old aRRR = 8.13, 95% CI = 2.40 to 27.54, vs. 18 to 25 years old respectively), to live in Manila (aRRR = 5.89, 95% CI = 2.20 to 15.72), exhibit hazardous drinking (aRRR = 2.87, 95% CI = 1.37 to 6.00) and problematic drug use (aRRR = 2.90, 95% CI = 1.21 to 7.13). HIV‐positive respondents were less likely to identify as straight (aRRR = 0.13, 95% CI = 0.02 to 0.72), and were more likely to avoid HIV services due to lack of anti‐lesbian, gay, bisexual and transgender (LGBT) discrimination policies (aRRR = 0.37, 95% CI = 0.14 to 0.90). Relative to HIV‐negative respondents, HIV‐unknown respondents were less educated (some college aRRR = 0.10, 95% CI = 0.02 to 0.37, beyond college aRRR = 0.31, 95% CI = 0.09 to 0.99, vs. high school or below respectively), had lower HIV knowledge (aRRR = 0.30, 95% CI = 0.20 to 0.71), and were less communicative about safer sex (ARR = 0.29, 95% CI = 0.09 to 0.92). Moreover, HIV‐unknown respondents were also more likely to have avoided HIV services due to cost (aRRR = 4.46, 95% CI = 1.73 to 11.52).

**Conclusions:**

This study highlights differences in HIV risks and healthcare engagement by HIV status. These findings show different barriers exist per HIV status group, and underscore the need to address Filipinx trans‐WSM and cis‐MSM’s poor engagement in HIV services in the Philippines.

## 1 INTRODUCTION

The HIV epidemic in the Philippines has become a national public health crisis, with diagnosed cases rapidly increasing by more than 500%, from 15,000 cases in 2010 to 77,000 in 2018 [1, 2]. Recent data suggest that Filipinx (gender neutral term for natives of the Philippines) transgender women and cisgender men who have sex with men (trans‐WSM and cis‐MSM respectively) account for four in five new HIV diagnoses annually [1, 3]. Efforts to increase HIV testing among these communities have remained low [3, 4], leaving many community members untested and unaware of their HIV status [5]. Surveillance estimates show that only 15% of trans‐WSM and 16% of cis‐MSM in the country have ever received HIV testing and know their HIV status [3, 6]. In addition, Filipinix trans‐WSM and cis‐MSM have been shown to engage in HIV‐risk behaviours such as drug/alcohol use, condomless sex and experience social marginalization (e.g. homelessness, engagement in sex work, unemployment) [7, 8, 9].

Designing effective HIV prevention/treatment programmes for trans‐WSM and cis‐MSM populations requires accounting for the unique needs per HIV status group [10, 11]. For example studies suggest that tailoring interventions is necessary for HIV‐positive cis‐MSM given the unique psychosocial stresses they face [12], including experiencing HIV‐related discrimination [13]. Additionally, research suggests that HIV‐positive cis‐MSM report more condomless sex [14], use of alcohol/drugs during sex more often [15] and lack sexual communication skills [16] compared to HIV‐negative cis‐MSM. Comparisons of HIV risk and healthcare engagement among people who are unaware of their HIV status (HIV unknown) is underexamined in the literature. One study that has examined this, however, found no significant predictors of sexual risks among HIV‐unknown individuals compared to HIV‐positive and ‐negative individuals [16]. These relationships are less known among trans‐WSM populations. Among a handful of studies that have documented barriers to healthcare engagement among trans‐WSM and cis‐MSM populations, barriers identified include discrimination by healthcare workers [17, 18], clinic wait time/location inconvenience [19, 20], and concerns about disclosing HIV status [21]. However, there remains limited research that compares experiences of healthcare engagement by HIV status among trans‐WSM and cis‐MSM populations [10, 11].

To date, there are no studies that investigate Filipinx cis‐MSM and trans‐WSM’s HIV risk behaviours and healthcare engagement experiences specifically by HIV status [22]. Such information is highly relevant given the Philippines has recently revamped its omnibus HIV law, Republic Act No. 11166 (RA 11166) [23]. Along with the Philippines’ national HIV plans [2, 24], the new law includes new directives for increasing coverage of HIV services among these two populations, including working with HIV/AIDS community‐based organizations (CBOs) to increase HIV testing efforts. Given the lack of existing knowledge of Filipinx trans‐WSM and cis‐MSM populations, there is a need for formative studies to investigate how these groups’ risk behaviours and healthcare engagement experiences may differ by HIV status. Such research would be informative for HIV programmes seeking to effectively optimize scale up of HIV testing programmes and other HIV services in limited resourced settings like the Philippines. Given the current national epidemiological data identifying trans‐WSM and cis‐MSM as highly vulnerable to HIV infection [1, 3], we conducted an exploratory study to examine the predictors of HIV risk and healthcare engagement factors by HIV status.

## 2 METHODS

### 2.1 Study design

The #ParaSaAtin study was a cross‐sectional online study that investigated the structural, social and behavioural factors that impact uptake of HIV/AIDS prevention services in the Philippines. Data collection occurred between June 2018 and August 2019. The study was conducted in Manila and Cebu, two metropolitan areas with highest HIV rates. Our analyses focused on characterizing correlates of HIV status among Filipinx trans‐WSM and cis‐MSM. Recruitment was done online using social media platforms (e.g. Facebook groups, Twitter) of local HIV/AIDS CBOs.

We utilized best‐practices for implementing online surveys [25]. We installed a “captcha box” into the survey to rule out non‐human survey takers or robots [26]. We also systematically implemented a cross‐validation programme that blocked any IP addresses that were not unique, which prevented multiple responses from the same individual [27, 28]. Any duplicated IP address were blocked from taking the survey.

Participants were eligible if they were in the following criteria: (1) 18+ years old, (2) identified as a trans‐WSM or cis‐MSM, (3) had condomless anal sex with a male partner in the past year, (4) were currently residing in Manila or Cebu, (5) gave written consent for participation and (6) demonstrated English comprehension by answering a brief online screening survey composed of true/false questions based on the consent form. After completion of the survey, all participants received a P300 (approximately $5.85 USD) electronic prepaid mobile load card.

### 2.2 Measures

#### 2.2.1 Outcome: self‐reported HIV status

The outcome for this study was self‐reported HIV status, which was assessed by asking participants whether they had ever received a reactive HIV test result (i.e. positive), a non‐reactive HIV test result (i.e. negative) or do not know their HIV status (i.e. unknown).

We analysed correlates of HIV status using respondents’ socio‐demographic factors, experiences of social marginalization, HIV‐risk and healthcare engagement indicators and history of alcohol and substance use behaviour.

#### 2.2.2 Socio‐demographics

With exception to sexual orientation, we adapted socio‐demographic items from the Philippines National Demographic and Health Survey [29]. Age was grouped into the following categories: 18 to 24, 25 to 29, 30 to 34, 35 or more years. Gender was self‐reported (i.e. transgender woman or cisgender man). Current residence was reported as either Manila or Cebu. Education level was categorized as follows: high school or below, some college and college or beyond. Annual income was grouped as follows: less than P10,000, P10,000 to P20,000, P20,000 to P30,000, P30,000 or more or no income. Sexual orientation was assessed by asking participants if they would describe themselves as gay/lesbian, bisexual, straight or another sexual orientation not listed.

#### 2.2.3 Social marginalization

For social marginalization, we asked respondents if they had ever experienced homelessness (*yes/no*), had engaged in sex work within the past four months (*yes/no*), and/or were currently unemployed (*yes/no*).

#### 2.2.4 HIV risk and other healthcare engagement indicators

We asked respondents a series of yes/no questions about their healthcare engagement experiences including whether they had current health insurance and had avoided HIV services due to any of the following: cost, distance to/from healthcare facility, sexual orientation, gender identity and lack of anti‐discrimination policy related to being lesbian, gay, bisexual and/or transgender (LGBT). These items were based on our formative qualitative work [9, 10]. We also assessed participants’ HIV knowledge using the International AIDS Questionnaire (Cronbach alpha = 0.85, median = 42, range = 23 to 85), and dichotomized scores into high versus low HIV knowledge at the median [30]. We assessed respondents’ HIV sexual risk by asking how consistently they used condoms in the past year (i.e. *never/occasionally vs. always*), utilization of condoms at last sexual encounter (*yes/no*) and if they engaged in safer sex communication with their partners (*never communicated vs. have communicated*).

#### 2.2.5 Alcohol and substance use

Using the Alcohol Use Disorders Identification Test‐Concise (AUDIT‐C, Cronbach alpha = 0.83, median = 4, range = 0 to 14), we categorized any respondents with a score of greater than or equal to 4 as exhibiting hazardous drinking [31]. Based on the standard cut‐off score, we categorized respondents scoring greater than 1 point for problematic drug use in the Drug Use Disorders Identification Test consumption questions (DUDIT‐C, Cronbach alpha = 0.85, median = 1, range = 0 to 13) [32].

### 2.3 Statistical analysis and approach

Proportion and χ^2^ tests were performed to provide descriptive statistics. Factors associated with HIV status were investigated using binominal and multinomial logistic regression procedures. Co‐variates included demographics, experiences of social marginalization, HIV risk, healthcare engagement and alcohol/ substance use severity. We then tested for effect modification by gender identity. Prior to our final multinomial logistic regression model, we tested for multicollinearity by assessing the variance inflation factor (VIF), and determined that there was no collinearity in our data (all VIFs < 4.00) [33]. We used StataSE version 15.1 [34] for all analyses with alpha set at *p* < 0.05.

### 2.4 Ethical approval

Study procedures were deemed appropriate by our three study partners (PinoyPlus, Positive Action Foundation Philippines Incorporated and The Association for Transgender People in the Philippines – local HIV/AIDS CBOs who contributed to the development of the survey). The study protocol was approved by the Brown University Human Research Protection Program Institutional Review Committee.

## 3 RESULTS

### 3.1 Respondent characteristics

A total of 318 participants were participated in this study, of which 139 (44%) identified as transgender women and 179 (56%) identified as cisgender men. A total of 121 (38%) of the respondents were self‐reported having HIV‐negative status, 107 (34%) having HIV‐positive status and 90 (28%) having HIV‐unknown status. (See Table 1).

**Table 1:** Respondent characteristics (n = 318) by self‐reported HIV status

	Total n (%^c^)	Self‐reported HIV status			χ^2^ *p*‐value
		HIV negativen (%^c^)	HIV positiven (%^c^)	HIV unknownn (%^c^)	
Total	318 (100.00)	121 (38.05)	107 (33.64)	90 (28.31)	
Demographics					
Age					
18 to 24	85 (27.60)	41 (35.34)	13 (12.62)	31 (35.83)	<0.001
25 to 29	113 (36.69)	40 (34.48)	46 (44.66)	27 (30.34)	
30 to 34	56 (18.18)	24 (20.69)	21 (20.39)	11 (12.36)	
35+	54 (17.53)	11 (9.48)	23 (22.33)	20 (22.47)	
Gender					
Trans‐WSM	139 (43.71)	54 (44.63)	36 (33.64)	49 (54.44)	0.013
Cis‐MSM	179 (56.29)	67 (55.37)	71 (66.36)	41 (45.56)	
Current living location					
Cebu	63 (19.81)	40 (33.06)	11 (10.28)	12 (13.33)	<0.001
Manila	255 (80.19)	81 (66.94)	96 (89.72)	78 (86.67)	
Education					
High school or below	78 (24.61)	15 (12.50)	23 (21.50)	40 (44.44)	<0.001
Some college	57 (17.98)	27 (22.50)	23 (21.50)	7 (7.78)	
College or beyond	182 (57.41)	78 (65.00)	61 (57.01)	43 (47.78)	
Past year income					
Less than P10,000	74 (23.27)	23 (19.01)	21 (19.63)	30 (33.33)	0.002
P10,000 to P20,000	65 (20.44)	22 (18.18)	30 (28.04)	13 (14.44)	
P20,000 to P30,000	41 (12.89)	18 (14.88)	14 (13.08)	9 (10.00)	
P30,000+	84 (26.42)	43 (35.54)	26 (24.30)	15 (16.67)	
No income	54 (16.98)	15 (12.40)	16 (14.95)	23 (25.56)	
Sexual orientation					
Gay/lesbian	172 (54.09)	65 (53.72)	63 (58.88)	44 (48.89)	0.012
Bisexual	89 (27.99)	32 (26.45)	34 (31.78)	23 (25.56)	
Straight	37 (11.64)	18 (14.88)	2 (1.87)	17 (18.89)	
Not listed	20 (6.29)	6 (4.96)	8 (7.48)	6 (6.67)	
Social marginalization factors					
Ever homelessness					
No	259 (81.70)	103 (85.12)	83 (78.30)	73 (81.11)	0.409
Yes	58 (18.30)	18 (14.88)	23 (21.70)	17 (18.89)	
Recent (<4 months) sex work engagement					
No	222 (69.38)	90 (74.38)	86 (80.37)	46 (50.00)	<0.001
Yes	98 (30.63)	31 (25.62)	21 (19.63)	46 (50.00)	
Currently unemployed					
No	105 (33.02)	26 (21.49)	37 (34.58)	42 (46.67)	<0.001
Yes	213 (66.98)	95 (78.51)	70 (65.42)	48 (53.33)	
HIV risk & other healthcare engagement indicators					
Current health insurance					
No	182 (57.23)	57 (47.11)	54 (50.47)	71 (78.89)	<0.001
Yes	136 (42.77)	64 (52.89)	53 (49.53)	19 (21.11)	
Avoided HIV services due to cost of services					
No	176 (55.35)	81 (66.94)	68 (63.55)	27 (30.00)	<0.001
Yes	142 (44.65)	40 (33.06)	39 (36.45)	63 (70.00)	
Avoided HIV services due to distance of travel to/from healthcare facility					
No	180 (56.60)	71 (58.68)	69 (64.49)	40 (44.44)	0.015
Yes	138 (43.40)	50 (41.32)	38 (35.51)	50 (55.56)	
Avoided HIV services due to sexual orientation					
No	204 (64.15)	83 (68.60)	84 (78.50)	37 (41.11)	<0.001
Yes	114 (35.85)	38 (31.40)	23 (21.50)	53 (58.89)	
Avoided HIV services due to gender identity					
No	207 (65.09)	83 (68.60)	83 (77.57)	41 (45.56)	<0.001
Yes	111 (34.91)	38 (31.40)	24 (22.43)	49 (54.44)	
Avoided HIV services due to lack of LGBT anti‐discrimination policy					
No	216 (67.92)	88 (72.73)	89 (83.18)	39 (43.33)	<0.001
Yes	102 (32.08)	33 (27.27)	18 (16.82)	51 (56.67)	
HIV knowledge					
Low	176 (55.00)	51 (42.15)	52 (48.60)	73 (79.35)	<0.001
High	144 (45.00)	70 (57.85)	55 (51.40)	19 (20.65)	
Consistent condom use					
Never/occasionally	257 (80.31)	86 (71.07)	85 (79.44)	86 (93.48)	<0.001
All the time	63 (19.69)	35 (28.93)	22 (20.56)	6 (6.52)	
Condom use at last sex					
No	184 (57.50)	57 (47.11)	57 (53.27)	70 (76.09)	<0.001
Yes	136 (42.50)	64 (52.89)	50 (46.73)	22 (23.91)	
Safer sex communication					
Never communicated	59 (18.44)	13 (10.17)	16 (14.95)	30 (32.61)	<0.001
Have communicated	261 (81.56)	108 (89.26)	91 (85.05)	62 (67.39)	
Alcohol and substance use					
Hazardous drinking					
No	158 (49.38)	60 (49.59)	70 (65.42)	28 (30.43)	<0.001
Yes	162 (50.62)	61 (50.41)	37 (34.58)	64 (69.57)	
Problematic drug use					
No	226 (70.62)	101 (83.47)	71 (66.36)	54 (58.70)	<0.001
Yes	94 (29.38)	20 (16.53)	36 (33.64)	38 (41.30)	

χ^2^, chi‐square significance test.

At the time of the survey, most respondents were between ages 25 and 29 years old (113, 37%), currently lived in Manila 255 (80%), had a college education or beyond (182, 57%), made P30,000 or less in the past year (234, 74%), and identified their sexual orientation as gay/lesbian (172, 54%). A total of 58 (18%) had ever experienced homelessness and 98 (31%) had engaged in sex work in the past four months. About two‐thirds (213, 67%) of the sample was unemployed.

Most of the participants did not have health insurance (182, 57%). Respondents reported multiple reasons for avoiding HIV services including cost (45%), travel distance to/from healthcare facility (138, 43%), sexual orientation (114, 36%), gender identity (111, 35%) and lack of anti‐ LGBT discrimination policies at healthcare facilities (102, 32%). A total of 144 (45%) respondents had high HIV knowledge: 63 (20%) consistently used condoms, 136 (43%) used condoms during their most recent sexual encounter and 261 (82%) communicated safer sex to sexual partners. About half of the respondents exhibited hazardous drinking (162, 51%) and nearly a third displayed problematic drug use (94, 29%).

### 3.2 Relative risks of HIV‐positive respondents versus HIV‐negative respondents

Relative to HIV‐negative respondents, HIV‐positive respondents were more likely to be older, live in Manila, exhibit hazardous drinking and exhibit problematic drug use. Specifically, relative to 18 to 24 years old, older respondents had higher relative risk of reporting HIV‐positive status than HIV‐negative status (aRRR: 5.08, 95% CI 1.88 to 13.72; aRRR: 4.11, 95% CI 1.34 to 12.58; aRRR: 8.13, 95% CI 2.4 to 27.54 respectively). Compared those living in Cebu, those living in Manila were nearly six times more likely to report being HIV positive (aRRR = 5.89, 95% CI = 2.20 to 15.72). Relative to those who did not exhibit hazardous drinking, respondents exhibiting hazardous drinking were nearly three times more likely to report being HIV positive (aRRR = 2.87, 95% CI = 1.37 to 6.00). Finally, when compared to those without problematic drug use respondents with problematic drug use were nearly three times more likely to report being HIV positive (aRRR = 2.90, 95% CI = 1.21 to 7.13). See Table 2.

**Table 2:** Results of multinomial regression of self‐reported HIV status (HIV negative as base outcome)

	Self‐reported HIV status (base outcome: HIV negative)							
	HIV positive				HIV Unknown			
	Unadjusted RRR (95% CI)	*p*‐value	Adjusted RRR (95% CI)	*p*‐value	Unadjusted RRR (95% CI)	*p*‐value	Adjusted RRR (95% CI)	*p*‐value
Demographics								
Age								
18 to 24	Ref		Ref		Ref		Ref	
25 to 29	3.62 (1.70, 7.7)	0.001	5.08 (1.88, 13.72)	<0.001	0.89 (0.45, 1.75)	0.742	1.27 (0.48, 3.34)	0.621
30 to 34	2.75 (1.17, 6.49)	0.020	4.11 (1.34, 12.58)	0.013	0.60 (0.25, 1.42)	0.250	1.30 (0.40, 4.26)	0.656
35+	6.59 (2.54, 17.07)	<0.001	8.13 (2.40, 27.54)	<0.001	2.40 (1.01, 5.74)	0.048	3.04 (0.84, 10.96)	0.088
Gender								
Trans‐WSM	Ref		Ref		Ref		Ref	
Cis‐MSM	1.58 (0.92, 2.72)	0.091	1.17 (0.50, 2.73)	0.702	0.67 (0.38, 1.16)	0.159	2.03 (0.79, 5.27)	0.137
Current living location								
Cebu	Ref		Ref		Ref		Ref	
Manila	4.30 (2.07, 8.94)	<0.001	5.89 (2.20, 15.72)	<0.001	3.20 (1.56, 6.56)	0.001	1.50 (0.56, 4.01)	0.417
Education								
High school or below	Ref		Ref		Ref		Ref	
Some college	0.55 (0.23, 1.30)	0.178	0.40 (0.11, 1.43)	0.164	0.09 (0.03, 0.26)	<0.001	0.10 (0.02, 0.37)	0.001
College or beyond	0.51 (0.24, 1.06)	0.071	0.44 (0.13, 1.45)	0.180	0.20 (0.10, 4.16)	<0.001	0.31 (0.09, 0.99)	0.048^i^
Past year income								
Less than P10,000	Ref		Ref		Ref		Ref	
P10,000 to P20,000	1.49 (0.66, 3.35)	0.330	2.34 (0.78, 6.96)	0.125	0.45 (0.18, 1.08)	0.076	0.80 (0.24, 2.63)	0.719
P20,000 to P30,000	0.85 (0.34, 2.12)	0.731	0.60 (0.18, 1.99)	0.406	0.38 (0.14, 1.01)	0.052	0.79 (0.20, 3.10)	0.744
P30,000+	0.66 (0.30, 1.42)	0.292	0.35 (0.11, 1.03)	0.058	0.26 (0.12, 0.59)	0.001	0.44 (0.13, 1.44)	0.179
No income	1.16 (0.46, 2.93)	0.740	0.67 (0.17, 2.63)	0.575	1.17 (0.50, 2.74)	0.708	0.55 (0.14, 2.10)	0.391
Sexual orientation								
Gay/lesbian	Ref		Ref		Ref		Ref	
Bisexual	1.09 (0.60, 1.98)	0.762	0.92 (0.42, 1.98)	0.832	1.06 (0.54, 2.05)	0.858	0.61 (0.23, 1.58)	0.315
Straight	0.11 (0.02, 0.51)	0.005	0.13 (0.02, 0.72)	0.020	1.39 (0.64, 2.99)	0.394	1.36 (0.42, 4.42)	0.605
Not listed	1.37 (0.45, 4.19)	0.575	1.42 (0.32, 6.16)	0.635	1.47 (0.44, 4.87)	0.522	0.55 (0.14, 2.10)	0.477
Social marginalization factors								
Ever homelessness								
No	Ref		Ref		Ref		Ref	
Yes	0.63 (0.31, 1.24)	0.185	1.18 (0.42, 3.34)	0.743	0.75 (0.36, 1.55)	0.439	0.59 (0.21, 1.65)	0.317
Recent (<4 months) sex work engagement								
No	Ref		Ref		Ref		Ref	
Yes	0.70 (0.37, 1.32)	0.283	0.46 (0.16, 1.27)	0.136	2.90 (1.62, 5.17)	<0.001	1.04 (0.39, 2.76)	0.922
Currently unemployed								
No	Ref		Ref		Ref		Ref	
Yes	1.93 (1.07, 3.48)	0.028	2.42 (0.91, 6.44)	0.076	3.19 (1.75, 5.82)	<0.001	1.99 (0.71, 5.60)	0.190
HIV risk & other healthcare engagement indicators								
Current health insurance								
No	Ref		Ref		Ref		Ref	
Yes	0.87 (0.51, 1.47)	0.613	1.47 (0.67, 3.21)	0.324	0.23 (0.12, 0.44)	<0.001	0.51 (0.21, 1.23)	0.138
Avoided HIV services due to cost of services								
No	Ref		Ref		Ref		Ref	
Yes	1.16 (0.67, 2.00)	0.591	2.22 (0.94, 5.26)	0.068	4.72 (2.62, 8.51)	<0.001	4.46 (1.73, 11.52)	0.002
Avoided HIV services due to distance of travel to/from healthcare facility								
No	Ref		Ref		Ref		Ref	
Yes	0.78 (0.45, 1.33)	0.369	0.50 (0.21, 1.18)	0.117	1.77 (1.02, 3.08)	0.041	0.44 (0.17, 1.15)	0.096
Avoided HIV services due to sexual orientation								
No	Ref		Ref		Ref		Ref	
Yes	0.59 (0.32, 1.08)	0.093	0.36 (0.07, 1.80)	0.218	3.12 (1.77, 5.52)	<0.001	1.09 (0.23, 5.06)	0.912
Avoided HIV services due to gender identity								
No	Ref		Ref		Ref		Ref	
Yes	0.63 (0.34, 1.14)	0.130	1.44 (0.29, 7.05)	0.646	2.61 (1.48, 4.59)	0.001	1.35 (0.30, 6.01)	0.687
Avoided HIV services due to lack of LGBT anti‐discrimination policy								
No	Ref		Ref		Ref		Ref	
Yes	0.53 (0.28, 1.02)	0.061	0.37 (0.14, 0.90)	0.045	3.48 (1.95, 6.21)	<0.001	0.93 (0.32, 2.67)	0.984
HIV knowledge								
Low	Ref		Ref		Ref		Ref	
High	0.77 (0.45, 1.30)	0.329	0.81 (0.38, 1.75)	0.607	0.18 (0.10, 0.35)	<0.001	0.30 (0.20, 0.71)	0.006
Consistent condom use								
Never/occasionally	Ref		Ref		Ref		Ref	
All the time	0.63 (0.34, 1.17)	0.147	0.65 (0.25, 1.68)	0.382	0.17 (0.06, 0.42)	<0.001	0.46 (0.13, 1.67)	0.245
Condom use at last sex								
No	Ref		Ref		Ref		Ref	
Yes	0.78 (0.46, 1.31)	0.353	1.01 (0.44, 2.28)	0.976	0.27 (0.15, 0.50)	<0.001	0.43 (0.17, 1.07)	0.070
Safer sex communication								
Nevercommunicated	Ref		Ref		Ref		Ref	
Have communicated	0.68 (0.31, 1.49)	0.343	0.97 (0.31, 3.07)	0.971	0.24 (0.12, 0.51)	<0.001	0.29 (0.09, 0.92)	0.036
Alcohol and substance use								
Hazardous drinking								
No	Ref		Ref		Ref		Ref	
Yes	1.92 (1.12, 3.28)	0.016	2.87 (1.37, 6.00)	0.005	0.44 (0.25, 0.78)	0.005	0.75 (0.32, 1.75)	0.517
Problematic drug use								
No	Ref		Ref		Ref		Ref	
Yes	2.56 (1.37, 4.78)	0.003	2.90 (1.21, 7.13)	0.017	3.55 (1.88, 6.70)	<0.001	0.65 (0.67, 4.09)	0.272

RRR, relative risk ratios; ref, referent.

Relative to HIV‐negative respondents, HIV‐positive respondents were less likely to identify their sexual orientation as straight and avoided HIV services due to lack of anti‐LGBT discrimination policies. Specifically, the relative risk of reporting HIV‐positive serostatus decreased by a factor of 0.13 (aRRR = 0.13, 95% CI = 0.02 to 0.72) for straight respondents relative to gay/lesbian respondents. HIV‐positive respondents were less likely to report avoiding HIV services due to lack of anti‐LGBT discrimination policies (aRRR = 0.37, 95% CI = 0.14 to 0.90).

### 3.3 Relative risks of HIV‐unknown respondents versus HIV‐negative respondents

Analyses demonstrated that HIV‐unknown respondents were significantly disadvantaged. Relative to HIV‐negative respondents, HIV‐unknown respondents were less likely to have a college education (aRRR = 0.10, 95% CI = 0.02 to 0.37; aRRR = 0.31, 95% CI = 0.09 to 0.99 respectively). Moreover, HIV‐unknown respondents were less likely to have higher HIV knowledge (aRRR = 0.30, 95% CI = 0.20 to 0.71). Furthermore, HIV‐unknown respondents were less likely to have communicated safer sex with their partners compared HIV‐negative respondents (ARR = 0.29, 95% CI = 0.09 to 0.92). Moreover, relative to HIV‐negative respondents, HIV‐unknown respondents were more likely to have avoided HIV services due to cost (aRRR = 4.46, 95% CI = 1.73, 11.52).

## 4 DISCUSSION

To our knowledge, this is the first Philippines‐based study that provides formative empirical evidence of differences in HIV risk and healthcare engagement experiences among Filipinx trans‐WSM and cis‐MSM. Specifically, the results of this study expand the HIV literature in the Philippines by characterizing behaviours and health‐care experiences of these two communities stratified by HIV status. The present research provides valuable evidence for the development of HIV‐related public health programmes and policies designed for these populations. The authors recognize that trans‐WSM and cis‐MSM are distinct groups, both behaviourally and socially. However, our final adjusted model did not show significantly different relative risk of HIV by gender identity. As such, we report our primary outcome for both groups and did not stratify by gender. Our results emphasize the shared experiences of these two groups – namely the structural and social factors which result in elevated HIV risk. Therefore, our recommendations are not rooted in gender‐specific solutions, but in broader society level determinants of health behaviour that both groups share and are commonly subjected to.

Several factors influenced HIV status and care/prevention utilization. For instance older respondents were likely to report being HIV positive. This suggests there is a need to prioritize HIV treatment and prevention services by age and HIV status. We also found that a significant barrier to HIV treatment and prevention services was lack of anti‐LGBT discrimination policies. Unfortunately this is not surprising given the widespread discrimination and stigma faced by cis‐MSM and trans‐WSM [35, 36, 37]. Our formative qualitative research show that Filipinx trans‐WSM and cis‐MSM report negative healthcare experiences when receiving care from their HIV providers [9, 10]. Taken together, these results suggest that barriers to care may vary across age groups, which should be investigated in the future.

We also found that HIV‐positive respondents were more likely to identify as a sexual minority (e.g. gay/lesbian) and less likely to avoid HIV services due to discrimination within the healthcare sector. Although the HIV epidemic among trans‐WSM and cis‐MSM has become more dire over the past decade, only a small proportion of these high‐risk groups have sought out HIV prevention and treatment services [10]. Given that we relied on community‐based organizations for recruitment sampling, it is likely that these results reflect those who are HIV positive and are highly engaged in care, but may continue to experience discrimination based on their gender and sexual identities. While there is currently a national anti‐discrimination law (i.e. RA 11166) in the Philippines [38], it only protects individuals based on HIV‐positive status; there is no existing national law explicitly prohibiting discrimination based on gender or sexual identity [39]. This lack of protection may account for the observed gap in care utilization among respondents. Furthermore, there is a distinct paucity of HIV programmes in the Philippines for cis‐MSM and trans‐WSM. What limited resources are available may not provide culturally competent care specific to these groups. As such, our findings demonstrate a need for sensitivity training for healthcare providers and to prioritize culturally competent programmes that cater specifically to trans‐WSM and cis‐MSM [10].

Another important finding of this study is that HIV‐positive respondents exhibited higher alcohol/substance related problems compared to HIV‐negative participants, which is consistent with prior literature [40]. This finding is troubling given evidence that such alcohol/substance use problems are associated with suboptimal adherence to antiretroviral therapy [41]. This finding suggests integrating drug use harm reduction services within HIV care may improve health results [42]. These programmes may also allow patients to circumvent the discrimination they would normally experience in conventional healthcare settings (e.g. hospitals). That is such programmes can be designed to mollify the impact of stigma which impedes care access for trans‐WSM and cis‐.

One key difficulty to effective interventions to reduce problematic drug use in the Philippines is the current government’s War on Drugs. The current administration has enacted strict punitive measures in an attempt to control substance use [43]. Unfortunately this crackdown has driven people who use drugs away from harm reduction services [44]. It is likely that this has exacerbated substance use related issues among the population. As such, policymakers would do well to move away from harsh punitive measures and collaborate with community‐based organizations to promote safer alcohol/drug use. Such organizations may be particularly well‐suited as partners and leaders in implementing corrective interventions due to their having established trustworthy reputations among communities at risk, such as those living with HIV. Future intervention and policy development should continue to research how to work with these organizations to integrate substance use risk reduction and treatment services along with HIV care services.

We also found that participants who were HIV unknown had lower HIV knowledge, lower safer sex communication and higher avoidance of HIV services due to cost. These findings suggest that sexual education in the Philippines may be lacking. As such, it may be prudent for HIV programmatic goals to increase HIV testing outreach and engagement for community members who are unaware of their HIV status, particularly trans‐WSM and cis‐MSM. There have been recent initiatives in the Philippines to expand HIV testing opportunities, including engaging community health workers as HIV test counsellors and exploration of HIV self‐testing as a viable approach [45, 46]. In some areas in Metro Manila, “sundown” clinics have been established to offer HIV testing and counselling services in non‐stigmatizing settings and outside of typical workday hours [47]. Future efforts are needed to sustain these sundown clinics and support implementation of expanded HIV testing services in Metro Manila and elsewhere in the country. Programmes that provide information to trans‐WSM and cis‐MSM about HIV risk behaviours and that offer HIV testing/linkage services may improve HIV knowledge and engagement in care, a point for future research in the Philippines. In addition, more research is needed to better understand how to engage with high‐risk populations in the Philippines, including ways to potentially optimize the use of mobile technology to reach community members [48]. Special emphasis should go to improving current engagement as part of RA 11166’s implementation. Previous UNAIDS reports have suggested that only a quarter of trans‐WSM and cis‐MSM are covered by current HIV prevention programmes [2]. This suggests there is a need to develop educational interventions and use implementation methods that are relevant to meet trans‐WSM and cis‐MSM. Such work is particularly necessary to reach those who do not know their HIV status. This includes ensuring that HIV testing campaigns are designed to ease discomfort in trans‐WSM and cis‐MSM’s discussion of safer sex and provide explicit information on where they can receive HIV testing free of charge.

### 4.1 Limitations

There are several limitations to this study worth noting. First, due to the online convenience sampling strategy, the data only represent a portion of Filipinx trans‐WSM and cis‐MSM communities and cannot be generalized to all trans‐WSM and cis‐MSM in the Philippines. For example those who may live in areas outside the two metropolitan cities (i.e. rural community members) and those who are not linked to social media platforms employed in our recruitment efforts. Second, the cross‐sectional nature of these data does not permit causal inferences – we cannot say with certainty the directionality of the associations found. Lastly, the data were all self‐reported, and therefore may be inaccurate due to participants feeling uncomfortable disclosing their HIV status and personal behaviours/experiences. Moreover, since we were unable to verify participants’ HIV status, it is possible that participants’ self‐reported HIV statuses may have been inaccurate. Future studies should include biomarkers (i.e. confirmatory HIV testing) to determine actual HIV status.

The authors would like to make clear that although we did not find differences in our primary outcomes by gender identity, sexual risk behaviours and healthcare needs of trans‐WSM and cis‐MSM are demonstrably different. Although both groups are impacted by similar social and healthcare barriers (e.g. stigma and access) it would be incorrect to infer such barriers impact these groups identically. Our results shed light on the common healthcare barriers experienced by these groups, but it is beyond the scope of these data to make specific recommendations for each group separately. Future research investigating the specific gendered health outcomes (i.e. hormone use) as well as gendered social processes (e.g. transphobia, violence) of these groups in the context of the Philippines’ culture is necessary to develop tailored interventions for these two groups.

## 5 CONCLUSIONS

Our study is one of the first to characterize HIV risk behaviours and healthcare engagement experiences among Filipinx trans‐WSM and cis‐MSM by HIV status. Our results can inform future strategies to expand HIV testing programmes and treatment approaches in the Philippines. We found that relative to HIV‐negative respondents, HIV‐positive respondents were less likely to identify as straight and avoided HIV services due to lack of anti‐LGBT discrimination policies. In addition, HIV‐positive respondents were more likely to be older, live in Manila and exhibited hazardous drinking and problematic drug use. We also found that relative to HIV‐negative respondents, HIV‐unknown respondents were less educated, had lower HIV knowledge, were less communicative about safer sex, and were more likely to avoid HIV services due to cost. These findings demonstrate that members of different HIV status group encounter unique barriers to HIV prevention/care, and underscore the need to address Filipinx trans‐WSM and cis‐MSM’s poor engagement in HIV services. More work is needed to increase free HIV testing efforts among those trans‐WSM and cis‐MSM who remain untested and engage in high‐risk behaviours.

## COMPETING INTERESTS

The authors declare that they have no competing interests.

## AUTHORS’ CONTRIBUTIONS

AR, HJ, AO, AA, AS, LH, SC and DO were involved in the conceptualization of this paper. AR, SC and DO designed the analysis for this paper. AR conducted the data analysis. AR, HJ, AO, AA and DO analysed the data. AR wrote the paper. All authors have read, reviewed and approved the final version of this manuscript.

## ABBREVIATIONS

AUDIT‐C, Alcohol Use Disorders Identification Test‐Concise; cis‐MSM, cisgender men who have sex with men; DUDIT, Drug Use Disorders Identification Test consumption; LGBT, lesbian, gay, bisexual transgender communities; trans‐WSM, transgender women who have sex with men.

## References

[1] Joint United Nations Programme on HIV/AIDS (UNAIDS) . UNAIDS Data 2019. Geneva, Switzerland: Joint United Nations Programme on HIV/AIDS 2019 Available from: https://www.unaids.org/sites/default/files/media_asset/2019‐UNAIDS‐data_en.pdf (accessed 2019 Dec 10)

[2] National AIDS and STI Prevention and Control Program . HIV Prevention and Control Costed Operational Plan 2018–2020. 2017 Available from: https://www.aidsdatahub.org/sites/default/files/publication/Philippines_HIV_Health_Sector_Plan_2018‐2020_2017.pdf (accessed 2019 Dec 10)

[3] Philippines Department of Health . HIV/AIDS & ART Registry of the Philippines (HARP) Report – July 2019. Manila, Philippines: Department of Health National HIV/AIDS & STI Surveillance and Strategic Information Unit, Epidemiology Bureau. 2019 Available from: https://www.aidsdatahub.org/sites/default/files/publication/EB_HARP_July_AIDSreg2019.pdf

[4] United Nations Development Programme (UNDP) . Missing in Action – Loss of clients from HIV testing, treatment, care and support services: case studies of gay men and other men who have sex with men in Manila. United Nation Development Programme. 2017 Available from: http://www.ph.undp.org/content/philippines/en/home/library/hiv_aids/MissingInAction.html

[5] van Wijngaarden JWdL , Ching AD , Settle E , van Griensven F , Cruz RC , Newman PA . “I am not promiscuous enough!”: exploring the low uptake of HIV testing by gay men and other men who have sex with men in Metro Manila, Philippines. PLoS One. 2018;13:e0200256. 29979766 10.1371/journal.pone.0200256 PMC6034862

[6] Joint United Nations Programme on HIV/AIDS (UNAIDS) . UNAIDS Data 2018. Geneva, Switzerland: Joint United Nations Programme on HIV/AIDS 2018 Available from: https://www.unaids.org/sites/default/files/media_asset/unaids‐data‐2018_en.pdf 12349391

[7] Gangcuangco LMA , Tan ML , Berba RP . Prevalence and risk factors for HIV infection among men having sex with men in Metro Manila, Philippines. S Asian J Trop Med Public Health. 2013;44(5):810. 24437316

[8] Ross AG , Ditangco RA , Belimac JG , Olveda RM , Mercado ES , Rogers GD , et al. HIV epidemic in men who have sex with men in Philippines. Lancet Infect Dis. 2013;13(6):472–3. 23718916 10.1016/S1473-3099(13)70129-4

[9] Restar AJ , Adia A , Nazareno J , Hernandez L , Sandfort T , Lurie M , et al. Barriers and facilitators to uptake of condoms among Filipinx transgender women and cisgender men who have sex with men: a situated socio‐ecological perspective. Glob Public Health. 2020;15(4):520–31. 31630622 10.1080/17441692.2019.1679218 PMC7093239

[10] Restar A , Chan R , Adia A , Quilantang MI , Nazareno J , Hernandez L , et al. Prioritizing HIV services for transgender women and men who have sex with men in manila, Philippines: an opportunity for HIV provider interventions. J Assoc Nurses AIDS Care. 2020;34 (4):405–416. 10.1097/JNC.0000000000000131 PMC7257438 31592803

[11] Marks G , Crepaz N , Senterfitt JW , Janssen RS . Meta‐analysis of high‐risk sexual behavior in persons aware and unaware they are infected with HIV in the United States: implications for HIV prevention programs. J Acquir Immune Defic Syndr. 2005;39(4):446–53. 16010168 10.1097/01.qai.0000151079.33935.79

[12] Thompson SC , Nanni C , Levine A . The stressors and stress of being HIV‐positive. AIDS Care. 1996;8(1):5–14. 8664369 10.1080/09540129650125957

[13] Safren SA , O’Cleirigh C , Skeer MR , Driskell J , Goshe BM , Covahey C , et al. Demonstration and evaluation of a peer‐delivered, individually‐tailored, HIV prevention intervention for HIV‐infected MSM in their primary care setting. AIDS Behav. 2011;15(5):949–58. 20853023 10.1007/s10461-010-9807-8

[14] Van Kesteren NM , Hospers HJ , Kok G . Sexual risk behavior among HIV‐positive men who have sex with men: a literature review. Patient Educ Couns. 2007;65(1):5–20. 17098392 10.1016/j.pec.2006.09.003

[15] Purcell DW , Parsons JT , Halkitis PN , Mizuno Y , Woods WJ . Substance use and sexual transmission risk behavior of HIV‐positive men who have sex with men. J Subst Abuse. 2001;13(1–2):185–200. 11547619 10.1016/s0899-3289(01)00072-4

[16] Hays RB , Paul J , Ekstrand M , Kegeles SM , Stall R , Coates TJ . Actual versus perceived HIV status, sexual behaviors and predictors of unprotected sex among young gay and bisexual men who identify as HIV‐negative, HIV‐positive and untested. AIDS. 1997;11(12):1495–502. 9342072 10.1097/00002030-199712000-00014

[17] Ayhan CHB , Bilgin H , Uluman OT , Sukut O , Yilmaz S , Buzlu S . A Systematic review of the discrimination against sexual and gender minority in health care settings. Int J Health Serv. 2020;50(1):44–61. 31684808 10.1177/0020731419885093

[18] Wao H , Aluoch M , Odondi GO , Tenge E , Iznaga T . MSM's versus health care providers’ perceptions of barriers to uptake of HIV/AIDS‐related interventions: systematic review and meta‐synthesis of qualitative and quantitative evidence. Int J Sex Health. 2016;28(2):151–62.

[19] Dasgupta S , Kramer MR , Rosenberg ES , Sanchez TH , Reed L , Sullivan PS . The effect of commuting patterns on HIV care attendance among men who have sex with men (MSM) in Atlanta, Georgia. JMIR Public Health Surveil. 2015;1(2):e10. 10.2196/publichealth.4525 PMC4869235 27227128

[20] Colchero MA , Cortés‐Ortiz MA , Romero‐Martínez M , Vega H , González A , Román R , et al. HIV prevalence, sociodemographic characteristics, and sexual behaviors among transwomen in Mexico City. Salud Pública Méx. 2015;57(S2):99–106. 10.21149/spm.v57s2.7596 26545137

[21] Liu Y , Osborn CY , Qian H‐Z , Yin L , Xiao D , Ruan Y , et al. Barriers and facilitators of linkage to and engagement in HIV care among HIV‐positive men who have sex with men in China: a qualitative study. AIDS Patient Care STDs. 2016;30(2):70–7. 26784360 10.1089/apc.2015.0296 PMC4753635

[22] Restar A , Nguyen M , Nguyen K , Adia A , Nazareno J , Yoshioka E , et al. Trends and emerging directions in HIV risk and prevention research in the Philippines: a systematic review of the literature. PLoS One. 2018;13:e0207663. 30517178 10.1371/journal.pone.0207663 PMC6281194

[23] Congress of the Philippines . Republic Act No. 11166. Philippine HIV and AIDS Policy Act. 2018.

[24] Philippines National AIDS Council . 5th AIDS Medium Term Plan: 2011‐2016 Philippines Strategic Plan on HIV and AIDS. 2011 Available from: https://www.aidsdatahub.org/sites/default/files/documents/5th_AMTP_2011_2016_Philippines_NSP_April_29_2011.pdf (accessed 2019 Dec 10)

[25] Parsons C . Web‐based surveys: best practices based on the research literature. Visitor Stud. 2007;10(1):13–33.

[26] Baluni A , Gole S . Two‐step CAPTCHA: using a simple two step turing test to differentiate between humans and Bots. Int J Comput Appl. 2013;81(16):48–51.

[27] Van Selm M , Jankowski NW . Conducting online surveys. Qual Quant. 2006;40(3):435–56.

[28] King DB , O'Rourke N , DeLongis A . Social media recruitment and online data collection: a beginner’s guide and best practices for accessing low‐prevalence and hard‐to‐reach populations. Can Psychol. 2014;55(4):240–9.

[29] Philippine Statistics Authority . Philippines national demographic and health survey 2017. Quezon City, Philippines, and Rockville, MD: Philippine Statistics Authority and Inner City Fund; 2018.

[30] Davis C , Sloan M , Macmaster S , Hughes L . The international AIDS questionnaire—english version (IAQ‐E) assessing the validity and reliability. J HIV/AIDS Preven Child Youth. 2007;7(2):29–42.

[31] Bush K , Kivlahan DR , McDonell MB , Fihn SD , Bradley KA . The AUDIT alcohol consumption questions (AUDIT‐C): an effective brief screening test for problem drinking. Arch Intern Med. 1998;158(16):1789–95. 9738608 10.1001/archinte.158.16.1789

[32] Berman AH , Bergman H , Palmstierna T , Schlyter F . Evaluation of the Drug Use Disorders Identification Test (DUDIT) in criminal justice and detoxification settings and in a Swedish population sample. Eur Addict Res. 2005;11(1):22–31. 15608468 10.1159/000081413

[33] O’brien RM . A caution regarding rules of thumb for variance inflation factors. Qual Quant. 2007;41(5):673–90.

[34] StataCorp . StataSE 15.1 [computer software]. College Station, TX: StataCorp; 2018.

[35] Hughto JMW , Reisner SL , Pachankis JE . Transgender stigma and health: a critical review of stigma determinants, mechanisms, and interventions. Soc Sci Med. 2015;147:222–31. 26599625 10.1016/j.socscimed.2015.11.010 PMC4689648

[36] Poteat T , Wirtz AL , Radix A , Borquez A , Silva‐Santisteban A , Deutsch MB , et al. HIV risk and preventive interventions in transgender women sex workers. Lancet. 2015;385(9964):274–86. 25059941 10.1016/S0140-6736(14)60833-3 PMC4320978

[37] Sy W . Social determinants of HIV infection among men who have sex with men in the Philippines. 2016.

[38] Congress of the Philippines . Philippine HIV and AIDS policy Act: Republica act 11166. 2018.

[39] Adia AC , Restar AJ , Lee CJ , Payawal MP , Quilantang MI , Nazareno J , et al. Sword and shield: perceptions of law in empowering and protecting HIV‐positive men who have sex with men in Manila, Philippines. Glob Public Health. 2020;15:52–63. 31134838 10.1080/17441692.2019.1622762 PMC12273262

[40] Stein M , Herman DS , Trisvan E , Pirraglia P , Engler P , Anderson BJ . Alcohol use and sexual risk behavior among human immunodeficiency virus–positive persons. Alcohol Clin Exp Res. 2005;29(5):837–43. 15897729 10.1097/01.alc.0000164363.40533.e0

[41] Paolillo EW , Gongvatana A , Umlauf A , Letendre SL , Moore DJ . At‐Risk alcohol use is associated with antiretroviral treatment nonadherence among adults living with HIV/AIDS. Alcohol Clin Exp Res. 2017;41(8):1518–25. 28679147 10.1111/acer.13433 PMC5564671

[42] Haldane V , Cervero‐Liceras F , Chuah FL , Ong SE , Murphy G , Sigfrid L , et al. Integrating HIV and substance use services: a systematic review. J Int AIDS Soc. 2017;20:21585. 28692211 10.7448/IAS.20.1.21585 PMC5515016

[43] Tom Allard KL . Exclusive: 'Shock and awe' has failed in Philippines drug war, enforcement chief says. Reuters. 2019;1(1):1–1.

[44] Dombrowski JC , Dorabjee J , Strathdee SA . Atrocity in the Philippines: how Rodrigo Duterte's war on drug users may exacerbate the burgeoning HIV epidemic. J Acquir Immune Defic Syndr. 2017;76(1):23–25. 28797018 10.1097/QAI.0000000000001464 PMC5645061

[45] Sy TRL , Padmawati RS , Baja ES , Ahmad RA . Acceptability and feasibility of delegating HIV counseling and testing for TB patients to community health workers in the Philippines: a mixed methods study. BMC Public Health. 2019;19(1):185. 30760257 10.1186/s12889-019-6497-7 PMC6375216

[46] Gohil J , Baja ES , Sy TR , et al. Is the Philippines ready for HIV self‐testing? BMC Public Health. 2020;20(1):1–8. 31918706 10.1186/s12889-019-8063-8 PMC6953179

[47] Samonte GMJ , Navarro MN , Umali KAR , Zapanta MJ , Montevirgen NDS , Tiu JES , et al. Unearthing and addressing issues around linkage to care in the Philippines: findings from the SPADE intervention. Ann Epidemiol. 2017;27(8):518.

[48] Hemingway C , Baja ES , Dalmacion GV , Medina PMB , Guevara EG , Sy TR , et al. Development of a mobile game to influence behavior determinants of HIV service uptake among key populations in the philippines: user‐centered design process. JMIR Serious Games. 2019;7:e13695. 31859673 10.2196/13695 PMC6942189

